# Masked Polycythemia Vera and Iron Deficiency in a Fertile-Age Woman

**DOI:** 10.7759/cureus.33545

**Published:** 2023-01-09

**Authors:** Luís R Almeida, Diogo Faustino, Rita Gameiro, Vera Salvado, Luis Dias

**Affiliations:** 1 Internal Medicine, Centro Hospitalar Universitário Lisboa Central - Hospital de São José, Lisbon, PRT

**Keywords:** primary polycythemia, iron deficiency, chronic blood loss, jak2 mutation, masked polycythemia vera

## Abstract

Polycythemia vera (PV) is a myeloproliferative disorder that leads to increased red blood cell (RBC) mass. The V617F activating mutation for* Janus kinase 2* (JAK2) is a classic finding in PV, but it is not exclusive to this condition. The radionuclide assay is an accurate method for accessing RBC, but hemoglobin (Hb) and hematocrit (Htc) values are frequently the first abnormal markers reported in a routine blood count and the basis for further investigation. Diagnostic criteria for PV were recently updated to include lower thresholds for Hb and Htc, increasing diagnostic sensitivity. However, it has been reported that a subset of patients does not meet these thresholds, besides having an active masked disease. We are presenting a case of a fertile-age woman with menometrorrhagia, whose blood loss and consequent iron depletion worked as a limiting factor for Hb and Htc increase, delaying the proper diagnosis. Splenomegaly, iron deficiency markers, and low erythropoietin supported PV investigation. The correction of iron depletion led to the unveiling of covert erythrocytosis. Concomitant hemoglobinopathies and secondary causes for erythrocytosis were excluded. The diagnosis was confirmed with polymerase chain reaction (PCR) for V617F-JAK2 mutation and bone marrow biopsy. As this case highlights, despite not meeting diagnostic criteria at presentation, masked PV exhibited clinical, laboratory, and imaging features of active symptomatic disease. For that, a higher level of suspicion must be held for fertile-age women who present with normal Hb and Htc levels and significant iron depletion, in the presence of low serum erythropoietin or splenomegaly.

## Introduction

Polycythemia vera (PV) is a chronic proliferative neoplasm with clonal stem cell expansion of myeloid lineage. The core features include the amplification of red blood cell (RBC) mass and other myeloid cells, requiring differential investigation for other myeloproliferative disorders. The radionuclide assay is the gold standard method for accessing the RBC mass but it is not a routine exam and has limited accessibility to clinicians [1]. Therefore, hemoglobin (Hb) and hematocrit (Htc) are used as surrogate markers for RBC expansion, with wider availability and lower cost. The World Health Organization (WHO) and the British Society for Hematology updated the classification for myeloproliferative neoplasms (including PV) in 2016 and 2018, respectively [2,3]. According to the WHO guidelines, the diagnosis is now based on three major criteria: elevated Hb (> 16.5 g/dL in men and > 16 g/dL in women) or Htc levels (> 49% in men and > 48% in women) or increased RBC mass (> 25% above mean normal predicted value); bone marrow biopsy (BMB) with features of PV (hypercellularity for age, panmyelosis, and prominent erythroid growth); the presence of Janus kinase 2 (JAK2) V617F mutation (exon 14) or JAK2 exon 12 mutation (which together accounts for 98% of cases) [4,5]. Decreased serum erythropoietin level was introduced as a minor criterion for diagnosis when the first two major ones are verified [2].

Patients can experience symptoms such as aquagenic pruritus, fatigue, dizziness, nausea, upper abdominal discomfort, weakness, and peripheral paresthesia, which are not easily attributable to a single cause. PV is also associated with hyperviscosity, thrombosis, and bleeding, as well as a potential for progression to myelofibrosis and acute leukemia (blast phase) in about 20% of patients [5]. BMB is not mandatory for diagnosis but is the only exam to identify or predict progression [2,5].

Dysregulation of iron metabolism is also common in PV, as erythrocytosis contrasts with iron deficiency in a physiological paradox [6]. Iron deficiency is more frequent among women at a fertile age. In this group, chronic blood loss and iron depletion can work as limiting factors to the major RBC expansion observed in PV. This has diagnostic implications, as Hb and Htc levels cannot reach the defined PV diagnostic threshold, blurring the clinical picture and delaying a targeted investigation. This subset of patients has a masked PV, for which diagnosis is challenging [1-5].

We present the case of a fertile age woman with masked PV, where her menometrorrhagia-related iron deficiency masked the typical laboratory findings, despite having an active and symptomatic disease. This article was previously presented as a meeting poster at the 18^th^ European Congress of Internal Medicine on August 30, 2019.

## Case presentation

This is the case of a 41-year-old woman with a medical history of anxiety disorder and chronic anemia due to menometrorrhagia controlled with a combined oral contraceptive. She was also medicated with lisinopril 5 mg od owed to a recent diagnosis of arterial hypertension. She was admitted to the emergency department complaining of headache and dizziness, which was associated with a hypertensive crisis. Symptomatic treatment with captopril and metamizole was provided and she was discharged with a referral internal medicine outpatient appointment. On this follow-up consultation, a review of the presenting complaints revealed a history of fatigue, nausea and early satiety, holocranial pulsating headache (two to three times a week), dizziness, and acral paraesthesia involving wrists and hands occurring for the previous three months. Symptoms gradually became more frequent and disabling. At that time, under the mentioned contraception, the patient had regular menstrual cycles with a minimal flow lasting for four days. No other abnormal bleeding sources were reported. She denied chest pain or anginal equivalents, easy bruising, abdominal pain, jaundice, or pruritus. Physical examination revealed high blood pressure (158/85 mmHg) and a palpable spleen. Laboratory workup showed elevated RBC count and thrombocytosis, although with normal Hb and Htc. Profound microcytosis and hypochromia were confirmed in peripheral blood smear, and iron studies showed low levels of ferritin, serum iron, and transferrin saturation. The laboratory findings are summarized in Table 1.

**Table 1:** Laboratory investigation Initial laboratory workup and serial results after iron parental supplementation.

	Initial Results	Serial Results	Reference Range
Red Blood Cell Count	7.32	8.24	3.8 - 5.0 x 10^12^/L
Hemoglobin	13.6	15.8	12.0 - 15.0 x 10g/L
Hematocrit	44.6	50.6	35% - 46%
Mean Corpuscular Volume	60.9	62.9	78.0 - 96.0 fL
Mean Corpuscular Hemoglobin	18.6	18.9	26.0 - 33.0 pg
Reticulocyte Count	1.27	1.38	0.5 - 1.5%
White Blood Cell Count	8.15	6.78	4.5 - 11.0 x 10^9^/L
Platelet Count	625	783	150 - 450 x 10^9^/L
Ferritin	2.7	3.6	4.63 - 204.00 ng/mL
Serum Iron	19	20	50.0 - 170 ug/dL
Transferrin Saturation	4.4	4.7	20 - 40%
Erythropoietin	-	< 1.5	4.3 - 29.0 mUI/mL

**Figure 1:**
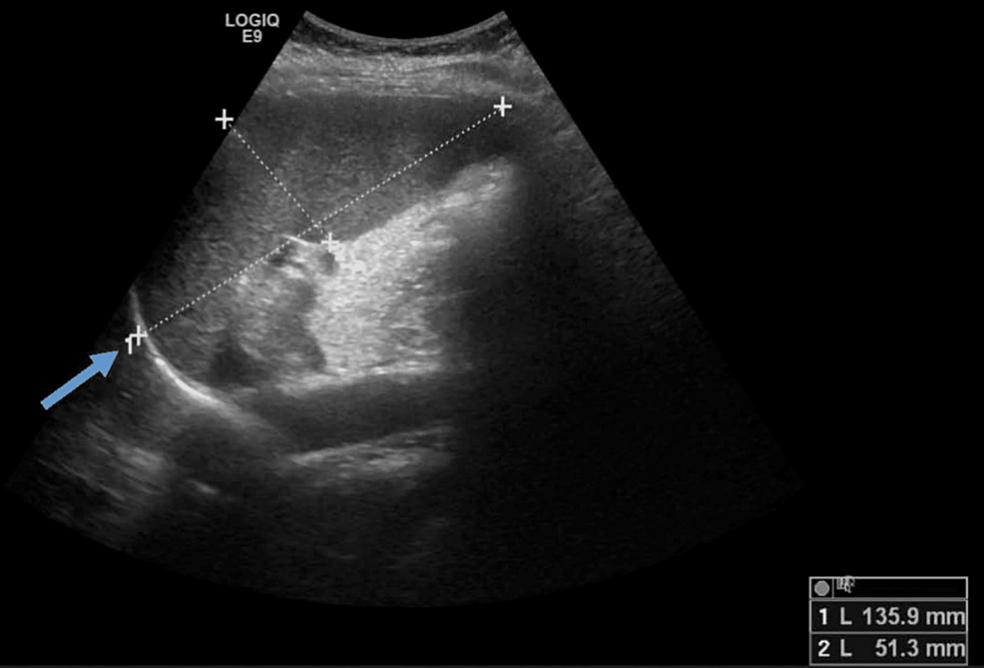
Splenomegaly on abdominal ultrasound Splenomegaly was confirmed in the abdominal ultrasound. The blue arrow identifies the splenic length axis of 135.9 mm.

Based on these results, parenteral iron supplementation was prescribed (iron-sucrose 1 gr). The antihypertensive treatment was switched to perindopril 5 mg od plus amlodipine 5 mg od, with effective blood pressure control on further ambulatory monitoring. After the iron parenteral boost, the patient described a transient improvement in her fatigue, but within weeks relapsed once again. Serial hemogram showed higher Hb and Htc but persistent low iron parameters and low serum erythropoietin below a measurable threshold (Table 1). Other causes for microcytosis and hypochromia such as thalassemia and secondary polycythemia were excluded. Splenomegaly was confirmed on abdominal ultrasound assessment (Figure 1).

PV was confirmed by the identification of the specific polymerase chain reaction for the V617F-JAK2 mutation. BMB revealed hypercellular medulla with mature trilineage growth, erythroid hyperplasia, and minor reticulin fibrosis, and it excluded dysplastic features. The patient started 100 mg of acetylsalicylic acid and was referred to a hematology appointment. Cytoreductive therapy with hydroxyurea was started, considering symptomatic disease, splenomegaly, and significant iron depletion (the patient was under contraception). At the one-year follow-up, hydroxyurea was titrated to 1 gr od, and the patient was clinically asymptomatic with a controlled blood count.

## Discussion

The diagnosis of masked PV is challenging. In the WHO 2016-revised diagnostic criteria for PV, a lower threshold for Hb and hematocrit was established aiming for an increase in diagnostic sensitivity and the inclusion of more suspected cases for workup. Despite these updates, masked PV cases have still been reported. Chronic blood loss is one of the features that can mask this condition [1,7]. In this case, a thorough history review, including menstrual cycle characteristics, and directed diagnostic workup and follow-up were required to unveil the disease.

Younger females are more prone to delayed diagnosis when compared to the mean diagnostic age in the sixth decade [8]. Even when symptoms are present, they can be misinterpreted or not recognized as part of the disease itself. In this case, the symptoms reported by the patient could be explained by iron depletion or arterial hypertension per se, blurring the major clinical picture, as the disease continued to develop. The assessment of spleen enlargement is a key point of the physical examination, which should be confirmed by abdominal ultrasound. This case also highlights the value of serum erythropoietin and RBC count, when PV is suspected and severe iron deficiency is present. These tests are widely available and can provide additional evidence to support PV investigation.

Masked PV patients are at higher risk of major complications and progression to overt myelofibrosis and acute leukemia [9]. Available treatment options have proven efficacy in reducing splenomegaly, symptoms, and thromboembolic events, which the latter represent the major cause of morbidity and mortality [10,11]. However, it has not been shown to prolong survival or reduce progression [11,12]. Aspirin and phlebotomies are the classic first line therapy in PV, although the latter tend to aggravate the iron deficiency, which can become intolerable for some patients. This fact is critical regarding patients with chronic blood loss.

Cytoreductive treatment is indicated based on the risk assessment, clinical findings, and symptoms [12]. The evidence suggests a better hematological and clinical response with cytoreductive therapy in masked PV patients [12,13]. Both hydroxyurea and pegylated interferon have proven efficacy in achieving sustained clinical response [12,14]. For fertile age women, preconception counseling is advised for a better choice of cytoreductive regimen, and regarding the risk of pregnancy complications. Hydroxyurea is teratogenic and should be used under continuous and reliable contraception methods, as was the case [12,15]. Pegylated interferon is a safe alternative for young women who are planning future pregnancies or not using reliable contraception [12,14,15].

As this case emphasizes, masked PV in women of reproductive age has proper characteristics on diagnostic and therapeutic pathways, making early recognition essential to appropriate management.

## Conclusions

Masked PV has an insidious presentation and unfulfilling laboratory criteria, despite the lower thresholds for Hb and Htc in the 2016 updated WHO guidelines. Fertile-age women are at higher risk of under or misdiagnosis, as well as patients with chronic bleeding sources and iron deficiency states. In this subset of patients, the presence of splenomegaly, low serum erythropoietin, or elevated RBC count is a key factor for raising suspicion of masked PV, despite normal Hb and Htc. Early diagnosis is essential for accurate risk assessment and management.
